# Highly Multifunctional GNP/Epoxy Nanocomposites: From Strain-Sensing to Joule Heating Applications

**DOI:** 10.3390/nano10122431

**Published:** 2020-12-05

**Authors:** Xoan F. Sánchez-Romate, Alejandro Sans, Alberto Jiménez-Suárez, Mónica Campo, Alejandro Ureña, Silvia G. Prolongo

**Affiliations:** Materials Science and Engineering Area, Escuela Superior de Ciencias Experimentales y Tecnología, Universidad Rey Juan Carlos, Calle Tulipán s/n, 28933 Móstoles, Madrid, Spain; a.sans@alumnos.urjc.es (A.S.); monica.campo@urjc.es (M.C.); alejandro.urena@urjc.es (A.U.); silvia.gonzalez@urjc.es (S.G.P.)

**Keywords:** carbon nanotubes, thermal properties, electrical properties, strain sensing, joule heating

## Abstract

A performance mapping of GNP/epoxy composites was developed according to their electromechanical and electrothermal properties for applications as strain sensors and Joule heaters. To achieve this purpose, a deep theoretical and experimental study of the thermal and electrical conductivity of nanocomposites has been carried out, determining the influence of both nanofiller content and sonication time. Concerning dispersion procedure, at lower contents, higher sonication times induce a decrease of thermal and electrical conductivity due to a more prevalent GNP breakage effect. However, at higher GNP contents, sonication time implies an enhancement of both electrical and thermal properties due to a prevalence of exfoliating mechanisms. Strain monitoring tests indicate that electrical sensitivity increases in an opposite way than electrical conductivity, due to a higher prevalence of tunneling mechanisms, with the 5 wt.% specimens being those with the best results. Moreover, Joule heating tests showed the dominant role of electrical mechanisms on the effectiveness of resistive heating, with the 8 wt.% GNP samples being those with the best capabilities. By taking the different functionalities into account, it can be concluded that 5 wt.% samples with 1 h sonication time are the most balanced for electrothermal applications, as shown in a radar chart.

## 1. Introduction

Nowadays, polymeric materials are gaining much attention. They present some interesting properties that make them suitable for use in a wide variety of applications, including as coatings for environmental and corrosion protection [1,2,3] or as a matrix in composite materials due to their high compatibility with most widely used reinforcements [4,5]. 

In this regard, the use of carbon nanoparticles such as graphene nanoplatelets (GNPs) or carbon nanotubes (CNTs) is now of interest. This can be explained by their excellent mechanical, thermal and electrical properties. In fact, they can reach values of Young’s Modulus over 1 TPa, thermal conductivity around 5000 W/mK and electrical conductivities of 10^7^ S/m [6,7,8,9,10]. These superior properties make them highly suitable for multiple applications. More specifically, they are commonly used as reinforcement for polymeric materials. Furthermore, when added to an insulator matrix, both electrical and thermal conductivity grow several orders of magnitude, becoming conductive materials, because of the creation of percolating networks inside the material [11,12,13]. These facts promote their use, for example, in Structural Health Monitoring (SHM), electromagnetic interference shield and as thermal interface materials (TIMs) [14,15,16,17,18,19].

This work is focused on the effect of GNP content and sonication time on several properties such as electrical and thermal conductivity, strain-sensing and Joule heating capabilities. Here, GNP nanocomposites have demonstrated good capabilities as strain sensors with gauge factors, that is, the correlation between the variation of the normalized electrical resistance divided by the applied strain is much superior to that of CNT-based ones, especially at higher strain levels [20,21,22,23,24,25]. This is explained by the 2D disposition of the GNPs within the material allowing a higher interparticle distance between adjacent nanoparticles, thus leading to a more prominent tunneling effect [26,27,28]. In addition, GNPs can be added into the resin in contents superior to those possible with CNTs without inducing a drastic degradation of mechanical properties. Therefore, these materials present much higher values of thermal conductivity, which mainly depends on nanofiller content [14,29,30]. 

However, the correlation among the different properties is sometimes not well understood, as there are multiple factors affecting the final properties of the nanocomposite, including content, dispersion and geometry of the nanofiller [11,24,31,32]. In this regard, several dispersion techniques are commonly used to ensure a proper homogenization of the nanofillers inside the polymer. Among others, three roll milling and sonication have proved to be the most effective techniques due to the higher shear or cavitation forces induced during the process, which lead to an adequate breakage of larger agglomerates along with some exfoliating mechanisms [33,34]. Moreover, the enhancement of one property can lead to the degradation of another, as observed in highly conductive nanocomposites that present low gauge factors, as the interparticle distance between adjacent nanoparticles is much lower [20]. For these reasons, this study aims to better understand the role of nanoparticle content and dispersion state in the final properties of the nanocomposites. 

First, the electrical and thermal conductivity of GNP nanocomposites are determined for different combinations of GNP content and sonication time. Then, two examples of specific applications are also measured and deeply explored: their use as strain sensors by means of electrical measurements with applied strain and their capacity for Joule’s effect resistive heating. Finally, a summary of the obtained results is shown by balancing the final properties of each material in order to select the optimum one as a function of the desired application. 

## 2. Experimental Procedure

### 2.1. Materials

The nanocomposite is based on a GNP reinforced epoxy matrix. The resin is an *Araldite LY 556* from *Hunstman* supplied by *Antala* (Barcelona, Spain) with an amino hardener *XB 3473* in a stoichiometry proportion of 100:23 monomer to hardener from the same supplier.

GNPs are *M25* supplied by *XG Sciences* (Lansing, MI, USA) with a lateral size of 25 μm and a thickness of 6 to 10 nm. 

### 2.2. Nanocomposite Manufacturing

First, GNPs were manually dispersed into the epoxy resin. The mixture was then subjected to an ultrasonication process by using a horn sonicator *UP400S* supplied by *Hielscher* (Teltow, Germany) at an amplitude of 80% and a pulse period of 0.5 s. Sonication time and GNP content were varied in order to analyze their effects in the final properties of the nanocomposites. Nanofiller content and sonication time were varied accordingly to that shown in Table 1. 

Once the dispersion was made, the mixture was degassed at 80 °C for 15 min in order to remove the possible entrapped air. Then, it was subjected to a curing cycle at 140 °C for 8 h.

Finally, the plates obtained were demolded and machined to the dimensions required by the different tests which are explained below.

### 2.3. Electrical, Thermal and Microstructural Characterization

**Figure 1:**
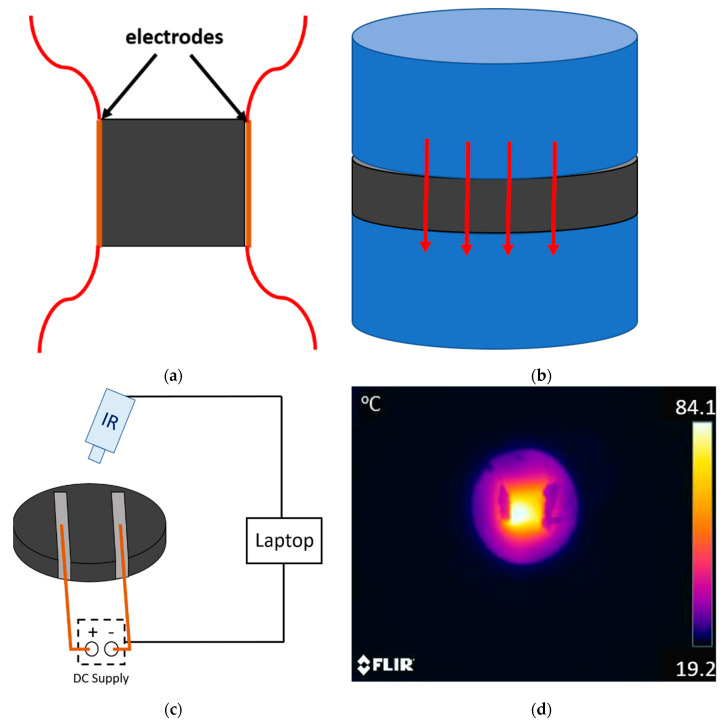
Schematics of (**a**) electrode disposition on the electrical conductivity tests, (**b**) set-up of thermal conductivity measurements (where the red arrows indicate the sense of the heat flow), (**c**) Joule heating tests indicating the electrode’s disposition in the sample and (**d**) an example of thermal image of a 5GNP-1 h sample (the dark shapes around the central region correspond to the silver paint coating of the electrodes).

Four-probe DC volume conductivity tests were carried out for 4 different samples of 10 × 10 × 1 mm^3^ dimensions for each condition (Figure 1a). Electrical resistance was determined as the slope of I-V curve, and electrical resistivity was determined accordingly to the geometry of the samples. The voltage range was set at 0–200 V for low conductive samples and 0–25 V for high conductive samples. The tests were performed in a *SMU, Keithley Instrument Inc. mod. 2410* (Cleveland, OH, USA).

Thermal conductivity was measured by estimating the heat flow through 50 mm diameter round samples by using a Heat Flow Meter (*FOX 50 Heat Flow Meter 190_C VHS 220VAC*) from *TA Instruments* (New Castle, DE, USA) as shown in the schematic of Figure 1b. The thickness of the samples varied in the range of 4–5 mm. Two samples were tested for each condition and thermal conductivity was determined at 30, 90 and 180 °C.

The GNP distribution was determined by means of Scanning Electron Microscopy analysis. For this purpose, GNPs were filtrated after sonication process in an acetone bath using a 0.22 μm porous paper. The obtained powder was then analyzed by using a *Hitachi S 3400N* apparatus from *Hitachi Global* (Tokyo, Japan).

### 2.4. Strain Monitoring Tests

Tensile tests were performed according to standard ASTM D638 at a test rate of 1 mm/min. Simultaneously, the electrical resistance was measured in order to characterize the strain monitoring capabilities of the manufactured materials by using an *Agilent* hardware *34410 A* (Agilent Technologies, Santa Clara, CA, USA). 

To achieve this purpose, the electrical resistance was recorded between two electrodes made of copper wire and silver ink to ensure a good electrical contact with the substrate. Here, the sensitivity, also called, gauge factor (*GF*) of the materials has been determined. 

*GF* is given by the ratio between the change of the normalized resistance divided by the applied strain:

(1) $$GF=\frac{\frac{\Delta R}{R_{0}}}{\varepsilon }$$

where $\Delta R/R_{0}$ denotes the change of the electrical resistance divided by the initial resistance of the specimen. 

In the tests conducted, *GF* was determined at low strain levels where crack mechanisms are not supposed to taking place. 

### 2.5. Joule Effect Heating Tests

Electrothermal properties were determined by resistive heating. In this experiment, thermal conductivity samples were subjected to a varying applied voltage. The temperature of the samples was measured by using a FTIR thermal camera (FLIR E50) (FLIR Systems, Wilsonville, OR, USA) as shown in the schematics of Figure 1c,d. The electrodes were also made with copper wire and silver ink with a distance of 30 mm between them. The voltage was applied by steps of 50 V until a temperature of around 180 °C was reached in the sample as it is near the degradation of the epoxy matrix. 

## 3. Results and Discussion

The electromechanical and thermal properties of GNP nanocomposites are discussed in this section. First, electrical conductivity measurements are shown, while thermal properties are also explored. Finally, the electromechanical characteristics of the proposed materials are given on the basis of strain monitoring tests. 

### 3.1. Electrical Properties of GNP/Epoxy Nanocomposites

**Figure 2:**
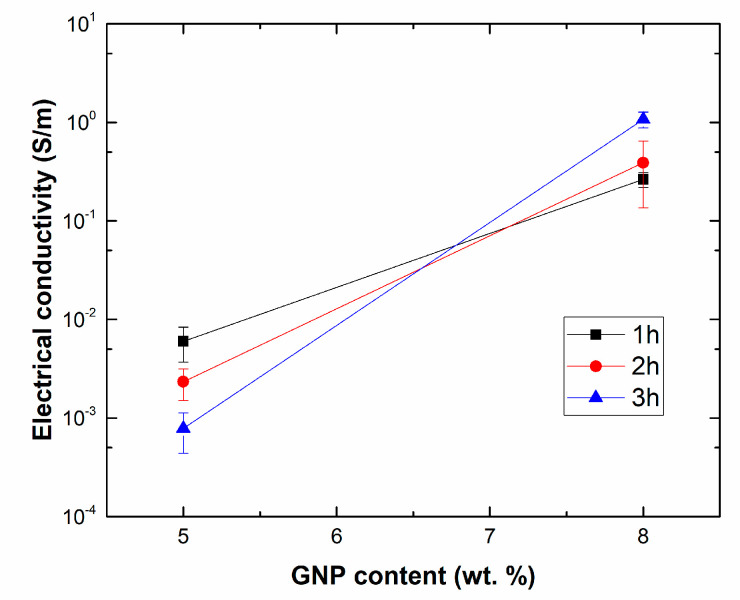
Electrical conductivity measurements for the different samples.

Figure 2 shows the values of the electrical conductivity for GNP nanocomposites. It can be observed that an increase of GNP content from 5 to 8 wt.% leads to a significant increase in the electrical conductivity, from values of 10^−4^–10^−3^ S/m to values of 0.1–1 S/m. This is easily explained by the effect of the higher volume fraction of the nanofillers that induces the creation of a higher number of percolating networks inside the material as has been widely explored in other studies [35,36].

However, the effect of sonication time is quite more complex. Here, at lower GNP contents, it is observed that the increasing sonication time leads to a reduction of the electrical conductivity. This is explained by the effect that sonication has on the GNP mixture. On one hand, there is the prevalence of exfoliating mechanisms of graphene layers during the sonication process [37], leading to a reduction of GNP thickness and, thus, to an increase of the aspect ratio of the nanoparticles as well as to an enhancement of GNP dispersion [38]. However, it has been also widely investigated that very large sonication times lead to a significant breakage of the nanofillers due to the higher cavitation forces induced during the sonication process [35]. This breakage of GNPs leads to a reduction in the lateral size. At lower contents, the viscosity of the media is low, so the cavitation forces are more effective [39]. This means that the optimum sonication time to achieve the best electrical performance is lower and this fact explains that increasing this sonication time too much could result in a detriment of the electrical properties, because of a very aggressive rupture of GNPs that leads to a reduction of the effective aspect ratio.

On the other hand, when increasing the GNP content, the viscosity of the mixture is much higher, so that cavitation process is not so efficient and the optimum sonication time to achieve the desired properties is increased. For this reason, the highest electrical conductivity for 8 wt.% GNP nanocomposites is achieved at 3 h of sonication time.

**Figure 3:**
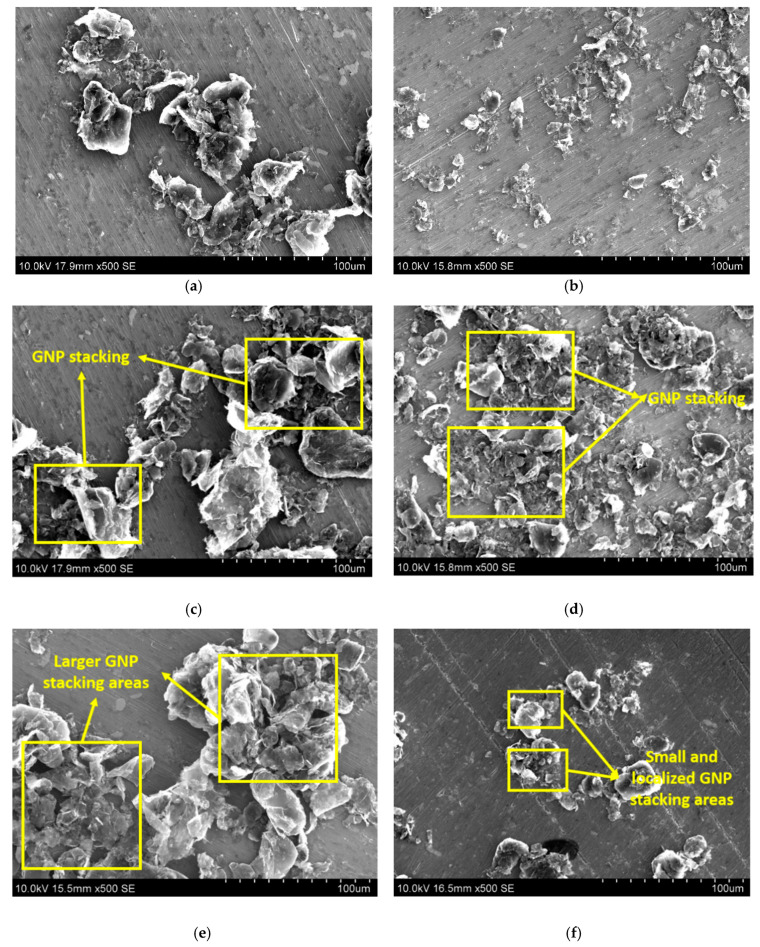
SEM images of GNP powder after the sonication process for (**a**,**c**) 5GNP-1 h, (**b**,**d**) 5GNP-3 h, (**e**) 8GNP-1 h, and (**f**) 8GNP-3 h samples.

In this regard, the SEM analysis of GNP powder after sonication process can confirm the previous statements. On one hand, when comparing 5GNP-1 h to 5GNP-3 h samples, an evident reduction of the lateral size can be pointed out (Figure 3a,b), which is more prevalent than the reduction of GNP stacking, which is qualitatively similar in both cases (Figure 3c,d) as, at low times, the sonication is effective at this GNP content. However, in the case of 8GNP samples, the increase of the sonication time from 1 h to 3 h promotes a very efficient reduction of the GNP stacking (Figure 3e,f) due to the previously commented higher efficiency of the sonication process at higher times explained by the higher viscosity of the mixture. 

These statements are of high novelty, as sonication time can have a positive effect on GNP properties depending on the viscosity of the mixture, as observed for 8 wt.% samples whereas longer sonication times will have a negative effect at lower contents due to an initial higher efficiency of the process that trends to rapidly break the nanoplatelets. 

### 3.2. Thermal Properties of GNP/Epoxy Nanocomposites

**Figure 4:**
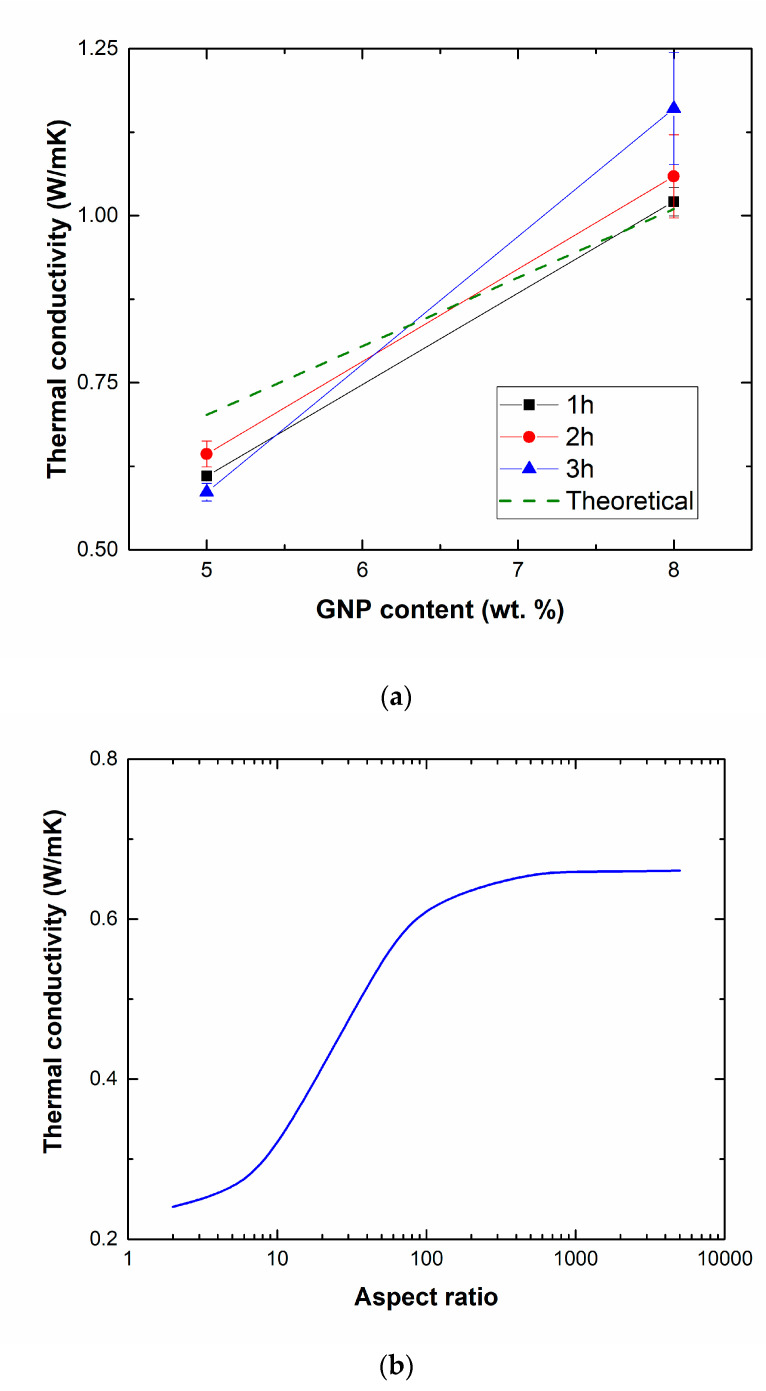

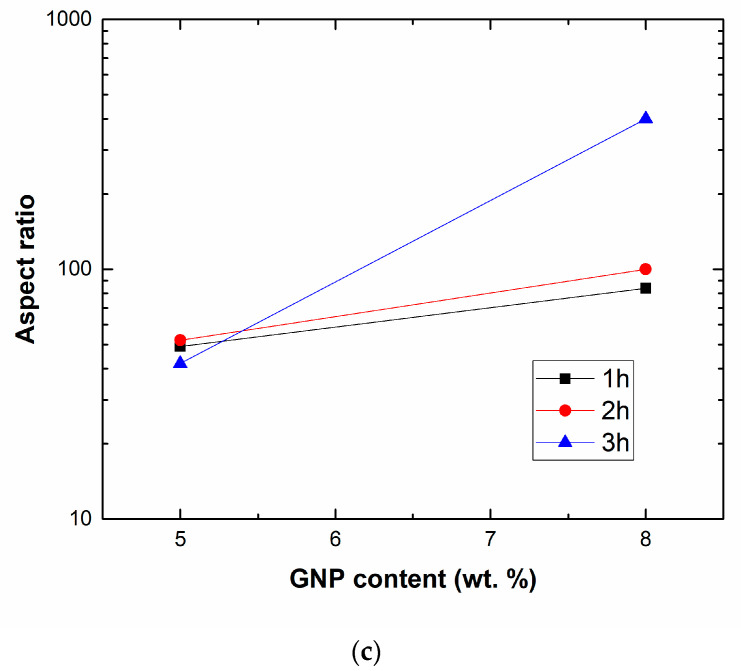
(**a**) Thermal conductivity measurements and theoretical estimations (dashed line), (**b**) variation of thermal conductivity for a 5 wt.% GNP sample accordingly to Hatta et al. model [40] as a function of GNP aspect ratio, and (**c**) estimation of aspect ratio accordingly to Hatta model for the different conditions.

Figure 4 summarizes the values of the thermal conductivity for GNP nanocomposites. It is observed that an increase of GNP content induces an enhancement of the thermal conductivity, as expected, due to a higher presence of nanofiller. Here, the effect of sonication time is not so prevalent, and only induces slight differences in the thermal properties of the nanocomposites. This can be explained on the basis of the role of GNP geometry and distribution inside the material. 

Thermal conductivity can be estimated from Hatta et al. model [40] knowing the thermal conductivity of the epoxy and the GNPs:

(2) S11=S22=β2×(β2−1)32×[β×(β2−1)12−cosh−1×β]S33= 1−2×S11

where β is the aspect ratio of GNPs, and *S*_11_, *S*_22_ and *S*_33_ are the thermal tensors in the principal axis. In the case of a 3D randomly distribution of nanofillers, the thermal conductivity of nanocomposite *k*_c_ can be estimated from the thermal conductivity of matrix, *k*_m_ (set as 0.22 W/mK) and from the nanoreinforcement *k*_f_ (set as 100 W/mK), as well as from its volume fraction, ϕ:

(3) $$\frac{k_{c}}{k_{m}}=1+\phi \times \left[\left(k_{f}-k_{m}\right)\times \left(2\times S_{33}+S_{11}\right)+3\times k_{m}\right]/J\;J=3\times \left(1-\phi \right)\times \left(k_{f}-k_{m}\right)\times S_{11}\times S_{33}+k_{m}\cdot \left[3\times \left(S_{11}+S_{33}\right)-\phi \times \left(2\times S_{11}+S_{33}\right)\right]+\frac{3\times k_{m}^{2}}{\left(k_{f}-k_{m}\right)}$$

Therefore, the aspect ratio of the nanofillers also plays a significant role. In this context, the dashed green line in Figure 4a indicates the estimation of the thermal conductivity when supposing that the aspect ratio of GNPs is the same for every condition. It can be observed that at lower contents the model generally overestimates the value of the thermal conductivity while at higher contents the estimations are below the measured values. This can be attributed to the differences in the geometry between GNPs for each condition. In this regard, the influence of the aspect ratio on the thermal conductivity is analyzed in the graph of Figure 4b. Here, an increase of the aspect ratio leads to an increase of the thermal conductivity, which is more prevalent in a range of l/d from 10 to 1000.

Therefore, the increasing thermal conductivity with sonication time in the case of 8 wt.% GNP nanocomposites is explained by the increase of the aspect ratio due to a better correlation between the exfoliation induced by cavitation forces and the breakage of GNPs and, thus, a reduction on the lateral size. The opposite effect is observed at lower contents, as explained previously, as the sonication process is much more aggressive and, thus, at higher sonication time there is a more prevalent breakage of GNPs in comparison to exfoliating effect. In this context, the graph of Figure 4c shows the prediction of the aspect ratio of GNPs by adjusting the theoretical model to the experimental measurements. Here, the reduction of the aspect ratio due to a very aggressive breakage of GNPs can be stated when increasing the sonication time at lower contents, whereas the opposite effect is clearly seen at higher contents, validating the previous statements. 

### 3.3. Analysis of Strain Monitoring Capabilities

**Figure 5:**
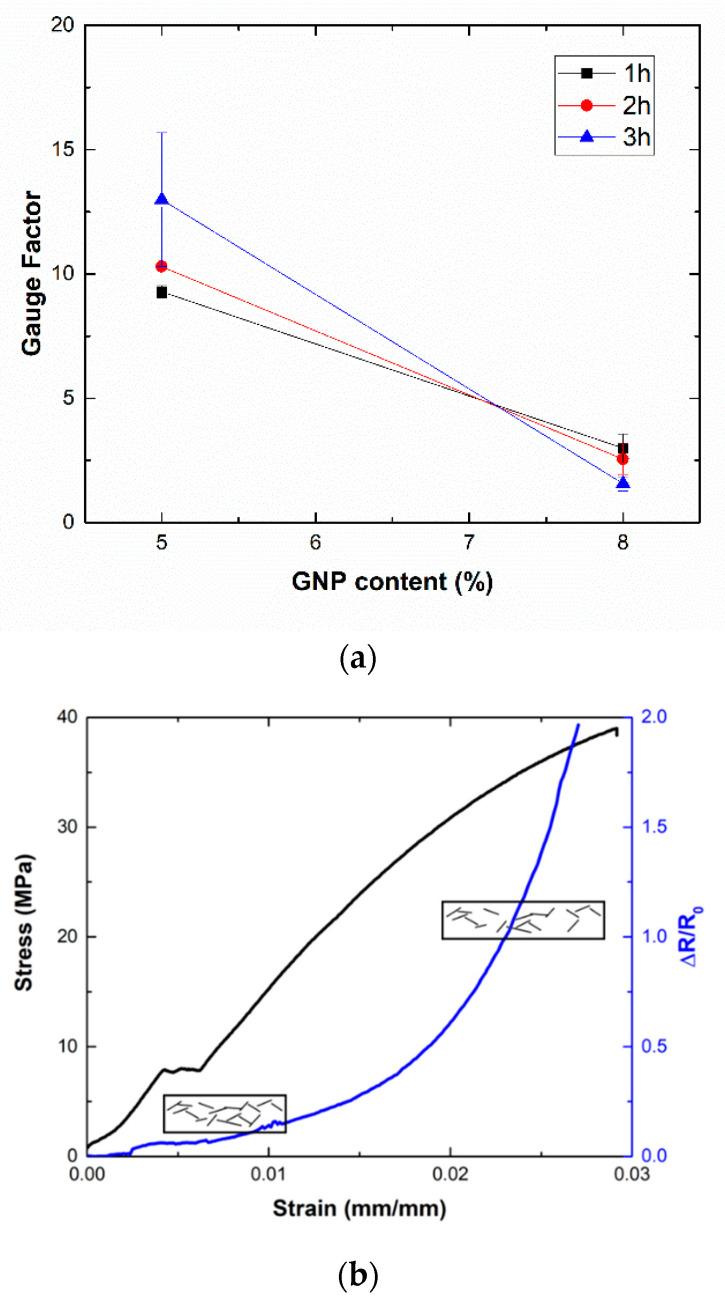
Electromechanic response of GNP nanocomposites showing (**a**) the measured GF and (**b**) an example of a strain-sensing curve.

Figure 5 summarizes the measured gauge factor at a low strain level (ε ~ 0.0025) for the different GNP nanocomposites. It can be noticed that GF values show the opposite trend when compared to the electrical conductivity measurements in Figure 2. This is explained by understanding the role of tunneling mechanisms inside the material. According to Simmons [41], the electrical resistance associated with tunneling mechanisms, $R_{tunnel}$, follows an exponential trend with the distance between adjacent nanoparticles, also called tunneling distance, *t*:

(4) $$R_{tunnel}=\frac{h^{2}t}{Ae^{2}\sqrt{2m\varphi }}exp\left(\frac{4\pi t}{h}\sqrt{2m\Phi }\right)$$

where *h* is Planck’s constant, *m* and *e* are the electron mass and charge, *A* the cross-sectional area of GNPs, and *φ* the height barrier of the matrix.

Therefore, the higher the tunneling distance, the more prevalent the exponential effect of tunneling resistance is. For this reason, lower values of conductivity, which imply higher values of tunneling resistance, usually lead to higher values of sensitivity, as seen in several studies [20,42].

Moreover, the electromechanical response of the samples shows a very prevalent exponential behavior, as can be seen in the graphs of Figure 5b. This is in good agreement with previous studies, where a prevalence of contact mechanisms takes places at a low strain level whereas the breakage of electrical pathways is dominant at higher strain levels, thus leading to a sharper increase of electrical resistance that is reflected in a higher GF at higher strain levels and, thus, to a very prevalent exponential response [24,42]. 

In this case, the effect of sonication time can significantly affect the sensing properties of these materials. At lower contents and due to a higher efficiency of the dispersion method, there is a reduction of the aspect ratio, as commented, that leads to an increase of percolation threshold [43,44]. This higher percolation threshold implies a higher distance between adjacent nanoparticles and thus, a higher sensitivity when increasing this time. However, the opposite effect can be clearly seen at higher contents, where the highest sensitivities are observed at the lowest sonication time. Therefore, to achieve the best sensing response, the system with lower GNP content and higher sonication time will be selected. 

### 3.4. Joule Effect Heating Analysis

**Figure 6:**
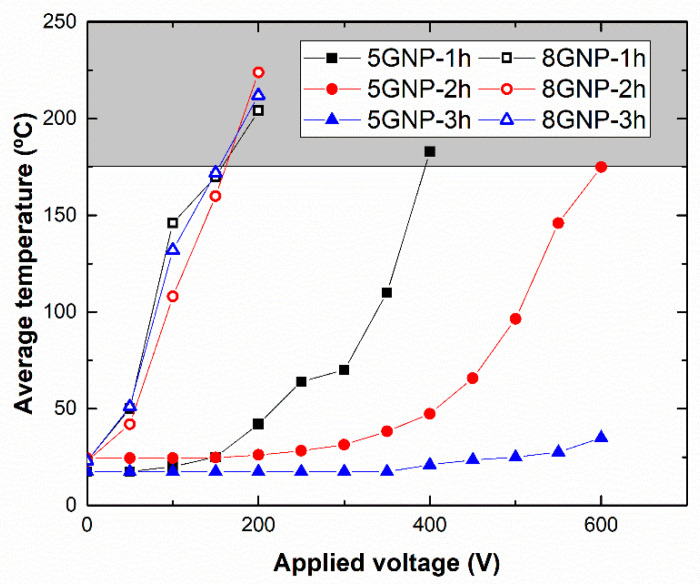
Average temperature reached as a function of the applied voltage for each tested condition (grey-colored area indicates the degradation zone of the epoxy resin).

Figure 6 summarizes the results of the Joule effect resistive heating tests, where the applied voltage and its corresponding average temperature reached in the sample are correlated. Here, it can be observed that both GNP content and sonication time have a significant influence in the resistive heating capacities of the samples. 

On one side, by increasing the GNP content, the average temperature reached on the samples increases drastically. More specifically, the maximum allowable temperature, given by the degradation temperature of the epoxy resin (around 180–200 °C), is reached at 400–600 V under the 5GNP-1 h and 5GNP-2 h conditions. However, for 8 wt.% GNP samples, the applied voltage needed is around 150–200 V.

On the other hand, the sonication time also affects the Joule heating properties of the nanocomposites. For 5 wt.% GNP samples, an increase in the sonication time implies a drastic decrease of resistive heating capabilities. In fact, samples with a sonication time of 1 h present a limit voltage of 400 V while samples with a sonication time of 3 h do not reached the maximum allowable temperature at the range of the voltage tested. Nevertheless, sonication time does not have a prevalent effect for the samples with an 8 wt.% GNP content, where the maximum allowable temperature is reached at a similar applied voltage. 

These results can be explained accordingly to Joule’s Law:

(5) $$Q=i^{2}\times R\times t$$

where *Q* is the generated heat during the test, *i*, the current flow, *R*, the electrical resistance of the specimen and *t* the time that the specimen is subjected to resistive heating. 

Therefore, the electrical properties of these materials play a crucial role in their resistive heating capabilities. Here, it can be concluded that the higher the electrical conductivity of the samples, the higher the current flow, *i*, and thus the heat generated during the Joule’s effect tests. This is in good agreement with the previously mentioned electrical conductivity results shown in Figure 2. For 5 wt.% GNP samples, there is a significant variation of electrical conductivity with sonication time which is reflected in a poor heating capability for the samples at 3 h of sonication. Moreover, in the case of 8 wt.% GNP samples, their higher electrical conductivity thus leads to higher heating properties. Here, the differences observed among the different sonication times are less prevalent as the electrical network formed inside the material is good enough to ensure proper electrical connections between adjacent nanoparticles. 

Furthermore, the Joule heating tests show very good heating capabilities in comparison to other studies with similar reinforcements and equivalent geometries [45]. Here, the main difference is correlated with the dispersion technique which, in the case of sonication, tends to form a more homogeneous dispersion inside the material without seriously affect the electrical and thermal properties of the GNPs themselves than in three roll milling process, where there is a prevalent breakage of GNPs due to the high shear forces involved in the dispersion process, leading to lower values of electrical and thermal conductivity and, thus, lower resistive heating capabilities. 

### 3.5. Analysis of Optimum Conditions for Application

**Figure 7:**
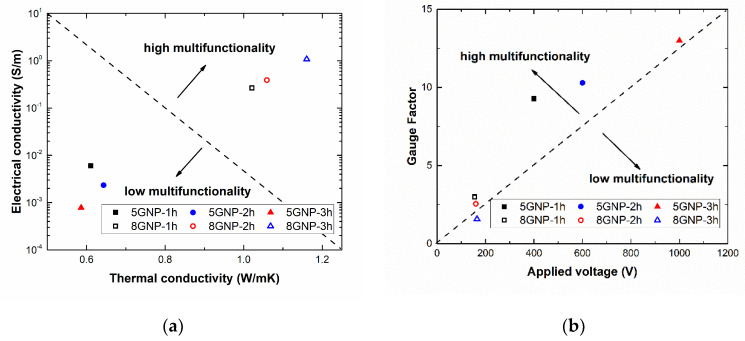
Graphs representing a comparison (**a**) between electrical and thermal conductivity and (**b**) between SHM capabilities, given by the Gauge Factor and Joule heating properties, given by the applied voltage to reach the maximum allowable temperature.

In this section, the behavior of the different manufactured samples is analyzed depending on the property tested. The aim is to select the optimum conditions depending on the desired application. In this context, Figure 7 shows a head-to-head comparison between the thermal and electrical conductivity (Figure 7a) and between SHM and Joule heating capabilities of the different samples (Figure 7b). 

In the first case, specimens with higher GNP contents and sonication times show the optimum combination of properties (hollow symbols of Figure 7a). This is explained by the prevalent role of the nanofiller content along with the selection of a higher sonication time that allows a more significant exfoliating effect without any substantial detriment on electrical and thermal properties of the GNPs themselves. However, when comparing the capability for SHM applications and Joule heating ones, the selection of an optimum condition is quite a bit more complex. This is explained by the opposite effect of Joule heating capabilities, which are mainly governed by the creation of a highly conductive electrical network inside the material and SHM ones, which are dominated by tunneling mechanisms that are more prevalent in less conductive networks. Here, 5GNP-1 h samples are very competitive (black solid symbol of Figure 7b), as they have a very high electrical sensitivity to strain due to a higher prevalence of tunneling mechanisms. In addition, their electrical conductivity is high enough to allow a relatively good Joule heating effect in comparison to 2 and 3 h samples because of a better GNP dispersion inside the material without affecting the intrinsic thermal and electrical properties, as previously explained. 

The selection of an optimum condition will therefore depend on the desired functionality. For these reasons, a radar chart was constructed to obtain a complete overview. 

In this chart, each measured property or functionality has been rescaled from 0 to 1, where 1 denotes the highest performance for this property. Therefore, the “best” material will have a factor of 1, whereas the rest of conditions were rescaled accordingly to their value of this property. 

This re-scalation follows a linear trend for Joule Effect, Gauge Factor and thermal conductivity. However, due to the highest sensitivity to small variations of electrical conductivity, it has been rescaled following a logarithmic trend, where 1 denotes again the highest measured conductivity and 0, the value of conductivity at percolation threshold, fixed at 10^−6^ S/m as observed in other studies [46,47].

**Figure 8:**
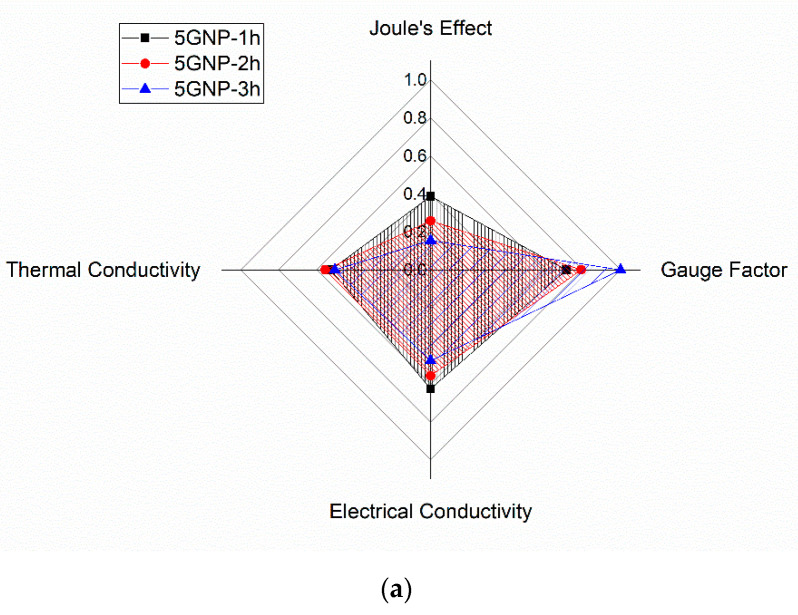

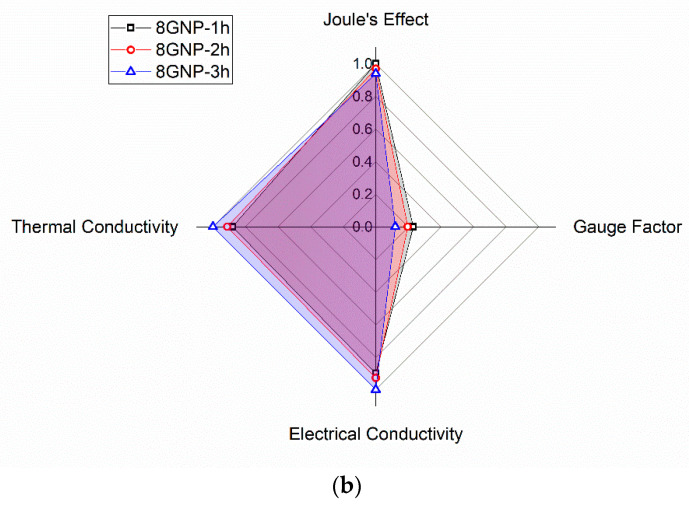
Radar chart of the different tested properties for (**a**) 5GNP and (**b**) 8GNP samples, scaled from 0 to 1.

Figure 8 shows the calculated values of the factors for each property and condition tested. Here, it can be observed that there is a high correspondence among electrical, thermal and Joule heating properties, whereas electrical sensitivity follows an opposite trend due to the previously commented factors. Accordingly, 5GNP-1 h seems to be a very promising solution for accomplishing all the analyzed functionalities, due to the good balance conferred by a good GNP dispersion without any detriment on nanoparticle intrinsic properties. More specifically, when compared to other works with similar nanoreinforcements, they show much higher Joule heating capabilities [45,48] and similar gauge factors at low strain levels [20,42], showing a high potential for diverse applications. 

## 4. Conclusions

Thermo-electrical and strain-sensing capabilities of GNP nanocomposites were deeply studied by varying GNP content and sonication time.

It was observed that the strain-sensing capabilities and the electrical conductivity follow an opposite trend. Here, the highest strain-sensing gauge factors have been achieved for the samples with the lower GNP content and higher sonication time, which show the lowest electrical conductivity. Furthermore, the effect of the dispersion procedure by means of sonication time on electrical and electromechanical properties was also explored. At lower GNP contents, higher sonication times induced a higher breakage of GNPs, with this effect being more prevalent than the exfoliating effect. However, at higher GNP contents and due to the higher viscosity of the mixture, the exfoliating effect is more prevalent at higher sonication times, explaining the higher values of electrical conductivity reached for these samples. 

Concerning the thermal and electrothermal properties, a similar trend to that of electrical conductivity is noticed. Here, the samples with higher GNP content show the highest thermal conductivities and Joule heating capabilities. However, dispersion procedure at higher contents does not play a crucial role, as there is a high enough percolating network to ensure good resistive heating responses. 

Therefore, by comparing the measured properties, it is possible to select the optimum manufacturing conditions as a function of the desired application. In this regard, 5GNP-1 h samples show a good balance among properties, as their Joule heating capabilities are much higher than 2 and 3 h sonication samples and their sensitivity is also much higher than 8 wt.% GNP specimens. Furthermore, they are very competitive when compared to similar nanocomposites of the literature. 

## Figures and Tables

**Table 1:** Nomenclature used for materials manufactured and tested.

GNP Content (wt.%)	Sonication Time (h)	Designation
5	1	5GNP-1 h
	2	5GNP-2 h
	3	5GNP-3 h
8	1	8GNP-1 h
	2	8GNP-2 h
	3	8GNP-3 h
